# The experiences of using polio outbreak simulation exercises to strengthen national outbreaks preparedness and response plans in sub-Saharan Africa

**DOI:** 10.11604/pamj.2020.36.340.23824

**Published:** 2020-08-25

**Authors:** Daudi Manyanga, Brine Masvikeni, Fussum Daniel

**Affiliations:** 1 WHO Inter-Country Support Team office for East and Southern Africa, P.O. Box 5160, Harare, Zimbabwe

**Keywords:** Polio outbreak, outbreak preparedness and response, risk assessment, wild polioviruses, circulating-vaccines-derived-polio viruses, simulation exercise

## Abstract

**Introduction:**

globally, by 2020 the paralytic poliomyelitis disease burden decreased to over 99% of the reported cases in 1988 when resolution 41.8 was endorsed by the World Health Assembly (WHA) for global polio eradication. It is clearly understood that, if there is Wild Poliovirus (WPV) and circulating Vaccines Derived Poliovirus (cVDPV) in the world, no country is safe from polio outbreaks. All countries remain at high risk of re-importation depending on the level of the containment of the types vaccine withdrawn, the laboratory poliovirus isolates, and the population immunity induced by the vaccination program. In this regard, countries to have polio outbreak preparedness and response plans, and conducting the polio outbreak simulation exercises for these plans remain important.

**Methods:**

we conducted a cross-section qualitative study to review to 8 countries conducted polio outbreak simulation exercises in the East and Southern Africa from 2016 to 2018. The findings were categorized into 5 outbreak response thematic areas analyzed qualitatively and summarized them on their strengths and weaknesses.

**Results:**

we found out that, most countries have the overall technical capacities and expertise to deal with outbreaks to a certain extent. Nevertheless, we noted that the national polio outbreak preparedness and response plans were not comprehensive enough to provide proper guidance in responding to outbreaks. The guidelines were inadequately aligned with the WHO POSOPs, and IHR 2005. Additionally, most participants who participated in the simulation exercises were less familiar with their preparedness and response plans, the WHO POSOPs, and therefore reported to be sensitized.

**Conclusion:**

we also realized that, in all countries where the polio simulation exercise conducted, their national polio outbreak preparedness and response plan was revised to be improved in line with the WHO POSOPs and IHR 2005. we, therefore, recommend the polio outbreak simulation exercises to be done in every country with an interval of 3-5 years.

## Introduction

Poliomyelitis is among the deadly and highly infectious vaccine-preventable (VPD) diseases, which cause paralysis mainly to the under-fives children caused by poliovirus serotypes 1,2, and 3 [1-3]. Globally there are two types of polioviruses, the vaccine poliovirus targeted for the global containment and wild poliovirus aimed for the eradication, yet the wild polio type 1 remained to be a challenge [4]. Globally there is tremendous progress towards polio certification as part of polio eradication, of the 6 World Health Organization (WHO) regions, only two are yet to be certified as polio-free, namely the WHO African Region and WHO Eastern Mediterranean Region [5]. Globally, the paralytic poliomyelitis disease burden decreased to over 99% of the reported cases in 1988 when the resolution 41.8 was endorsed by the World Health Assembly (WHA) for the global polio eradication [6-8]. The disease continued to spread to non-immune persons through fecal-oral and the virus affects nerves where approximately 0.5% of infected people present with acute flaccid paralysis which remains to be the gold standard for detecting and investigating polio outbreaks [1,2]. Afghanistan, Pakistan, and Nigeria are referred to be the only polio-endemic due to low population immunity against the disease. Even though there is tremendous progress toward polio certification of the remaining two WHO Regions and thereafter eradication, challenges still exist including operational in terms of funds, supplies, security, and technical challenges for containment [9-11].

It is clearly understood that, if there is Wild Poliovirus (WPV) and circulating Vaccines Derived Poliovirus (cVDPV) in the world, no country is safe from polio outbreaks [12,13]. As such, all countries remain at high risk of re-importation depending on the level of the containment of the types vaccine withdrawn, the laboratory poliovirus isolates, and the population immunity induced by the vaccination program [4,5]. The emergence of circulating Vaccines Derived Polio Virus type 2 (cVDPV2) in 2019 to 2019, 25 countries were involved globally of which 16 are WHO AFRO region increased operational challenge to polio eradication program due to increased immunity and surveillance gaps [14-16] Newly isolated vaccine-derived poliovirus, circulating vaccine-derived poliovirus, or wild poliovirus of any poliovirus serotype is a public health emergency and its implications can be devastating if not stopped its transmission with 120 days [17,18]. In this regard, poliomyelitis of wild poliovirus, or type 2 virus and vaccines derived poliovirus requires notification in all circumstances under the International Health Regulations(IHR) 2005. In line with the public health emergency and international health regulations, the Global Polio Eradication Initiative (GPEI) produced the Polio Outbreak Standard Operating Procedures (POSOPs) to enhance emergency polio event and outbreak responses which were endorsed by 68th WHA in May 2015 [7,8]. The two documents, POSOPs and IHR- 2005 guideline are used as benchmarks and gold standards for countries in responding to events and outbreaks in the light of experience gained by GPEI in handling outbreaks. The PSOPs continued to be produced with the regular revisions to stop polio outbreak or polio event with 120 days and suit the certification and post-certification goals towards global polio eradication [18-20]. On an annual basis, WHO set the condition that all countries as part of the certification process to compile and submit polio-free documentation to the African Region Certification Commission. The country full documentation is inclusive of updated preparedness and response plans for polio outbreaks risk mitigation in addition to containment plan which is in line with the updated version of the PSOPs and post-certification criteria [5,21].

**Polio outbreak risks assessment and risks mitigation plans:** polio outbreak is among the Public Health Emergency (PHE) diseases, in turn, the need of an outbreak preparedness and response plan is mandatory to all countries [22-24]. In the operation aspect, Sub-Saharan Africa (countries in the East and Southern Africa) considered the polio outbreak preparedness as the capability of the public health and health systems, communities, and individuals to prevent, protect against and quickly respond to and interrupt transmission within the 120 days. In this regard, the preparation of quarterly polio risk assessment and risk mitigation plans remained to be a regular activity for the prevention of the polio outbreak. The WHO AFRO region developed polio outbreak risks assessment guide which facilitates the assessment based on the Acute Flaccid Paralysis surveillance, population immunity for poliomyelitis, security level, and strengths of the vaccination program in assessment. Meanwhile, member states agreed to prepare and submit quarterly polio risk assessment and their risk mitigation plans. These are compiled by the WHO Inter-Country Support (IST)- Team for the East and Southern Africa (comprises of Botswana, Comoros, Eritrea, Eswatini, Ethiopia, Kenya, Lesotho, Madagascar, Malawi, Mauritius, Mozambique, Namibia, Rwanda, Seychelles, South Africa, South Sudan, Tanzania, Uganda, Zambia, and Zimbabwe).

In this study, we reviewed polio outbreaks (importation or emergence of wild poliovirus or circulating vaccine-derived poliovirus) risk assessments conducted by various organs including research articles involving countries in ESA from 2011 to 2019 and the grading of high, medium, and low risk as shown in Table 1 and Table 2 [25-30]. These polio outbreak risk assessments were among the key criteria used for prioritizing countries in conducting polio outbreak simulation exercises for the period in the East and Southern Africa sub-region. The main objective of conducting polio outbreak simulation exercises was to assess the capacity and level of preparedness of the Ministries of Health and key partners in responding to a polio event or an outbreak to contain it within 120 days as indicated in the POSOPs. Evaluation studies in Ethiopia, Madagascar and Nigeria have shown several gaps for the outbreak preparedness and response plans for emergencies in line with the IHR 2005, and therefore have limited ability to detect and respond to outbreaks [31]. Additionally, in Uganda for example an assessment of core capacities of IHR was conducted to 13 districts involving 61 health facilities, it was revealed that comprehensive preparedness plans incorporating IHR (2005) were lacking at national and district levels and most of health facilities (43%) had no surveillance guidelines [32,33]. In this regard, recommendations from polio simulation exercises were also aimed to improve the country national preparedness and response plan.

**Table 1:** the review of reported polio outbreaks risk assessment (importation or emerging of WPV or cVDPV) to Countries in the ESA sub-region ranked as high, medium, and low, 2017-2019

Year of Assessment	Countries at High risk (including medium high)	Countries at medium risk	Countries at lower risk	Organ and report of the assessment
2019	Ethiopia, Kenya, Mozambique, and South Sudan	Not explained	Botswana, Comoros, Eritrea, Eswatini, Lesotho, Madagascar, Malawi, Mauritius, Namibia, Rwanda, Seychelles, South Africa, Uganda, Tanzania, Zambia, and Zimbabwe	Global Polio Surveillance Status Report, 2019.Geneva: World Health Organization; 2019 (WHO/POLIO/19.08)
2019	Ethiopia, Kenya, Madagascar, Mozambique, South Sudan, and Uganda	Not explained	Botswana, Comoros, Eritrea, Eswatini, Lesotho, Madagascar, Malawi, Mauritius, Namibia, Rwanda, Seychelles, South Africa, Uganda, Tanzania, Zambia, and Zimbabwe	Polio End-Game Strategy 2019 -2023. Geneva: World Health Organization; 2019
2018	Kenya	Ethiopia, Madagascar, and South Sudan	Botswana, Comoros, Eritrea, Eswatini, Lesotho, Madagascar, Malawi, Mauritius, Namibia, Rwanda, Seychelles, South Africa, Uganda, Tanzania, Zambia, and Zimbabwe	Polio priority countries and the 2018 Hajj: Leveraging an opportunity (Research article)
2017	None	Ethiopia, Madagascar, South Africa, South Sudan, and Uganda	Botswana, Comoros, Eritrea, Eswatini, Lesotho, Kenya, Mozambique, Malawi, Mauritius, Namibia, Rwanda, Seychelles, Tanzania, Zambia, and Zimbabwe	An assessment of the geographical risks of wild and vaccine-derived poliomyelitis outbreaks in Africa and Asia (Research article)

**Table 2:** the review of reported polio outbreaks risk assessment (importation or emerging of WPV or cVDPV) to Countries in the ESA sub-region ranked as high, medium, and low, 2011-2014

Year of Assessment	Countries at High risk (including medium high)	Countries at medium risk	Countries at lower risk	Organ and report of the assessment
2014	Ethiopia, Kenya, South Sudan, and Uganda	Eritrea	Tanzania and Rwanda	Assessing and Mitigating the Risks for Polio Outbreaks in Polio-Free Countries-Africa, 2013-2014 (Research article)
2013	Ethiopia and Uganda	Kenya	Eritrea	Assessing the Risks for Poliovirus Outbreaks in Polio-Free Countries, Africa, 2012-2013 (Research article)
2011	Rwanda, Uganda, and Zambia	Not Explained	Not Explained	A Statistical Model of the International Spread of Wild Poliovirus in Africa Used to Predict and Prevent Outbreaks

## Methods

We conducted a cross-section qualitative study to review to 8 countries conducted polio outbreak simulation exercises in the East and Southern Africa from 2016 to 2018. The findings were categorized into 5 outbreak response thematic areas, analyzed qualitatively, and summarized them on their strengths and weaknesses. We reviewed the methodology and process used in preparing and conducting polio outbreak simulation exercises in the ESA countries, namely Eritrea, Ethiopia, Kenya, Malawi, Namibia, South Sudan, Tanzania, and Uganda from 2011 to 2019. The process includes reviewing all polio risk assessment reports for the countries and summarizes them. We collect final polio outbreak simulation exercise reports from Eritrea, Ethiopia, Kenya, Malawi, Namibia, South Sudan, Tanzania, and Uganda for a qualitative review based on the 5 thematic areas which were used in the reports. The thematic areas used in the review exercise were the same previously used for individual polio outbreak simulation exercises in countries. The thematic areas were as follows; the outbreak communication and coordination mechanisms, the initial outbreak response capacities, the quality of outbreak plans and documents, the flexibility of the outbreak plans and abilities, and the overall ability of the country to respond to an outbreak. The dummy tables were created for each thematic area and summarize the strength and weaknesses reported from each county conducted. The discussions were generated from the results and recommendations made for the future polio outbreak simulation exercise to come.

**Polio outbreak simulation exercises facilitators:** in all polio outbreak simulation exercises, a uniform standard of procedures and format was followed for the preparations and arrangements. The team facilitators were selected from the list of competent WHO surveillance officers, attended polio outbreak standard operating procedures training, and have practical experience in dealing with outbreaks. The selected facilitators were given documents from the country to be evaluated were shared with facilitators for perusal and in-depth familiarization at least two weeks before the real exercise. The documents shared were the national preparedness and response plans, standard polio outbreak operating procedures, investigation tools, reporting tools, social profiling tools, and immunization response planning tools. Additionally, facilitators arrived one day before the actual polio outbreak simulation exercise for task familiarization, conference facilities assessment, and rehearsal on the individual role.

**Polio outbreak simulation exercises participants:** participants were nominated by the individual countries according to their polio outbreak preparedness and response plans. The exercise expected that participants were involved in the development of the polio outbreak preparedness and response plan, and therefore knew the procedures involved and were able to simulate like real life. The list of participants involved the Ministries of Health, local government authority delegates, and local partners. In a situation where the country's national polio laboratory is within the country, the respective laboratory technologists were also involved.

**Polio outbreak simulation exercises:** in all polio outbreak simulation exercises, an introduction presentation was made to participants by facilitators including the polio eradication updates and prevailing risk for the country. The Ministries of health surveillance officers' in turns presents a summary of their national polio outbreak preparedness and response plans. Afterward, the facilitators briefly introduced the exercise and provide highlights on the ground rules of the exercise. The standard package of polio outbreak simulation exercises in ESA included seven progressive scenarios to be attended by participants in a real-life situation as stipulated in their polio preparedness and response activities in line with the WHO polio outbreak standard operating procedures. The duration for performing 7 standardized progressive scenarios for activities to be done in 120 days is only 16 hours (two days). All participants were given access to the internet, planning tools, information sheets, and simultaneous translation, while all facilitators are evaluating (objectively) using a standardized checklist. At the end of every progressive scenario, facilitators and participants discussed emerged issue. In this article, we review the key aspects of the conducted polio outbreak simulation exercises in ESA under the following thematic areas; outbreak communication and coordination mechanisms, initial outbreak response capacities, quality of outbreak plans and documents, the flexibility of the outbreak plans and abilities, and overall ability of the country to respond to the outbreak. We reviewed and summarize strengths and weaknesses identified in the exercises thematic areas. Additionally, we discussed recommendations from each simulation exercise that were related to the improvement or revision of the countries polio outbreak preparedness and response plans.

## Results

The polio outbreak simulation exercises were conducted in 8 countries from 2016 to 2019 based on the polio risk assessment results (Table 1 and Table 2). Some countries that were planned for polio simulation exercises had polio events or outbreaks including Madagascar, Mozambique, South Africa, and Zambia. In that regard, there was no need for conducting simulation exercises in those countries. The objectively scoring scale using a standardized checklist regarded that, any country reached at least 80% had a robust preparedness and response plan with minor improvement. So far for the period, only two countries achieved 80% and above target. The initial scenario in the polio outbreak simulation exercise involved communication from the polio laboratory indicating the country had a polio outbreak from one of the reported AFP cases investigated. Every country responded to the scene at different speeds, some were confused about what to do. Table 3 summarizes the individual country responses to this thematic area. The evaluators were expecting that immediately after the communication from the polio laboratory the national task force or any other body indicated in the national preparedness and response plan for the country will convene a meeting. Additionally, the national task force was expected to activate the polio outbreak subcommittees to execute their roles and responsibilities in responding to the outbreak. Furthermore, evaluators were also expecting communication from the national tasks force the subnational levels, media, and bordering countries as instructed by the IHR 2005. The initial outbreak response capacities scenario was prepared to evaluate how the country team was able to work on the subcommittees to conduct a detailed outbreak investigation and come up with a detailed investigation report which in real life is within 72 hours from the initial information. Furthermore, the subcommittees were expected to communicate with each other and develop a joint comprehensive initial outbreak response plan based on the national polio preparedness and response plan, WHO POSOPs, and IHR -2005. Most countries that conducted simulation exercises were responding based on their personal experiences with inadequate guidance from their national task force. Nevertheless, of the government leading the process in almost all countries, most plans were not aligned with WHO POSOPs. Additionally, standard planning templates were sometimes not used in the planning process. Table 4 below summarizes individual country performances in this thematic area.

**Table 3:** the outbreak communication and coordination mechanisms

Country	Observed strengths	Observed weaknesses
Eritrea	The communication from the polio laboratory (KEMRI) to the National Task Force was well simulated with an immediate set up for outbreak response.	The simulated national task force and the subcommittees were not aware of their roles and responsibilities in the simulation exercise, and no communication was made for neighboring countries as indicated in the IHR 2005.
Ethiopia	The roles and responsibilities for the outbreak coordination organs were well written in the national plan.	The simulated command post and participants were less aware of outbreak communication and coordination mechanism protocol written on the National polio outbreak preparedness and response plan
Kenya	The different immunization stakeholders involved in the initial communication of the polio outbreak response and participated in the simulation exercise.	There were no clear communication roles of key partners (WHO, UNICEF, etc) observed in the simulation exercise, and the outbreak was not declared as explained in the National polio outbreak preparedness and response plan, WHO POSOPs, and IHR-2005.
Malawi	The roles were written in the national polio outbreak response plan for communication and coordination mechanisms.	Participants simulated the plan were not familiar with their roles and responsibilities, and partners were inadequately engaged in the outbreak response scenario.
Namibia	Communication and coordination observed based on the individual participant's previous outbreak response experiences in the country context.	Reference of national polio outbreak response plan was inadequately done by participants while conducting the simulation exercise, and outbreak declaration was not done as indicated in IHR -2005 guideline.
South Sudan	Outbreak communication was done by the Ministry of Health high-level officials and, observed simulated crisis outbreak communication following the IHR-2005 guideline, and the POSOPs.	The National Preparedness and response plan did not show clearly committees TORs and responsibilities, and the organizations dealing with refugees, and internally displaced populations were inadequately engaged.
Tanzania	Observed various stakeholders were communicated in the simulation exercise and participated in the outbreak response activities.	The Ministry of Health management team participated in the outbreak simulation exercise did not follow the national polio outbreak response plan for the outbreak communication as well as the IHR- 2005 guideline.
Uganda	Observed communication and timely activation of the emergency operation center with clear roles and responsibilities for the stakeholders.	Observed delayed communication and coordination of the outbreak response subcommittees simulated teams with inadequate participation of the Ministry of Health's top decision-makers in the exercise.

**Table 4:** the initial outbreak response capacities

Country	Observed strengths	Observed weaknesses
Eritrea	The participated teams use the standard templates to identify key surveillance activities, vaccines, and requirements estimations and C4D activities.	The simulated outbreak response team failed to come up with an outbreak response plan aligned with the WHO POSOPs and IHR-2005 guidelines.
Ethiopia	Participants involved in the simulation exercise knew outbreak response, vaccination response, and outbreak communication	The simulated outbreak response was inadequately aligned with the WHO POSOPs and IHR-2005 guidelines.
Kenya	Observed Government commitment in the outbreak response processes and took a leadership role in all teams.	Limited coordination and communication made among the outbreak response working group in the simulation exercise in line with the WHO POSOPs.
Malawi	The simulated team developed an outbreak response plan with some surveillance enhancement and vaccination response activities	The developed outbreak response plan was not comprehensive (age group not defined, no special population consideration, no cold chain plan) and inadequately aligned with the WHO POSOPs and IHR -2005 guideline
Namibia	Observed outbreak response plan developed using a standard template with a timeline of activities	The simulated team prepared a general outbreak response plan with no focus to age group, special population, and vaccines/supplies requirements.
South Sudan	The initial outbreak response plan, risk assessment, and timeline of outbreak response activities for the simulated scenario were developed within the allocated time.	No clear guide was evidenced in the national polio outbreak preparedness and response plan for the initial outbreak response plan, outbreak risk assessment, and timeline of outbreak response activities.
Tanzania	The initial outbreak response plan with key elements on surveillance; advocacy, communication and social mobilization; and vaccination response was developed by the simulated team	The national task force inadequately communicated with the outbreak sub-working group in coming up with a joined country outbreak response plan according to the national polio outbreak response plan and IHR 2005.
Uganda	Initial outbreak response plan was developed timely in the simulation exercise with key activities for surveillance to enhance the plan, advocacy communication and social mobilization, and vaccination response	The initial outbreak response plan did not cover the vaccine withdrawal plan, environmental surveillance, and immediate vaccine request plan as directed in the WHO POSOPs.

Table 5 summarizes the quality of outbreak plans and documents in the conducted polio outbreak simulation exercises in the 8 countries. The team of evaluators expected the simulation team to come up with quality 6 months outbreak response plan with all essential components to mention a few, enhanced surveillance, C4D/communication, human resources, vaccination response, mOPV2 vaccine withdrawal, and Situation Reports (SITREP). Additionally, the prepared outbreak plans were expected by evaluators to have activity timelines as guided by WHO POSOPs for the period. In the review, no country managed to come up with detailed SITREP. However, Uganda and South Sudan managed to prepare at least quality outbreak response plans within the allocated time. The flexibility of the country outbreak preparedness and response plans was an important area that was assessed by the evaluators in the conducted polio outbreak simulation exercises. The specific country findings for this scenario were summarized in Table 6. In that scenario, a new polio outbreak information was communicated by the polio laboratory in addition to the initial outbreak. The country teams were expected to respond to two or more outbreaks simultaneously by adjusting the initial outbreak response plan. The adjustment of the initial outbreak preparedness and response plan was to be aligned with the WHO POSOPs and IHR 2005. We observed that only a few countries adjusted their outbreak response plans to accommodate emerged new outbreak, and some failed due to limited capacities. In the review of the overall ability of the country to respond to an outbreak as shown in Table 7, we realized only two countries South Sudan and Uganda managed to convince evaluators on their abilities and capacities to respond to a possible polio outbreak or polio event. Nevertheless, we observed from 8 countries reviewed that their national polio outbreak preparedness and response plans were not aligned with WHO POSOPs and IHR 2005. Additionally, the majority of participants in the conducted simulation exercises were less aware of their national polio outbreak preparedness and response plan especially the leaders. Not surprising, all countries reported to review and update their polio outbreak preparedness and response plan as a key recommendation.

**Table 5:** the quality of outbreak plans and documents

Country	Observed strengths	Observed weaknesses
Eritrea	An outbreak response plan development process was observed involving various subcommittee.	No polio outbreak response plan was developed by the simulation team in the allocated time as expected.
Ethiopia	Developed an outbreak response plan with a list of outbreak response key activities	The prepared polio outbreak response plan in the simulation exercise was not comprehensive and the SITREP was not shared as expected.
Kenya	Developed an outbreak response plan and SITREP which were prepared in the allocated time for simulation exercise.	The polio outbreak plan was prepared without using the standard outbreak response plan templates.
Malawi	Developed an outbreak response plan with some outbreak response key elements and a standard template for the SITREP	The prepared outbreak response plan was not aligned with the WHO POSOPs, and SITREP was not shared as expected.
Namibia	Developed an outbreak response plan with surveillance enhancement, communication, and social mobilization activities.	The prepared polio outbreak response plan was not comprehensive and inadequately aligned with the WHO POSOPs.
South Sudan	Developed a comprehensive outbreak response plan (covering the mobile population, addressing accessibility, and security challenges) in the allocated time.	The SITREP prepared had limited information for improved decision making towards outbreak response activities.
Tanzania	Developed an outbreak response plan and SITREP produced using standard templates in the simulation exercise.	Observed prepared combined simulated outbreak response plans were simulated based on the technical expertise and experiences of participants but not in line with their national polio outbreak preparedness and response plan.
Uganda	A six months outbreak response plan was developed and SITREP produced within the allocated simulation time	The plan developed was not comprehensive for all subcommittees, the WHO POSOPs were not followed, and special populations were not covered by the plan.

**Table 6:** the flexibility of the outbreak plans and abilities

Country	Observed strengths	Observed weaknesses
Eritrea	The team was able to modify the plan to accommodate new outbreak information and had a plan for human resource support (Surge A and B)	The observed existence of technical challenge to respond to a new outbreak from the previous outbreak response arrangement in line with the WHO POSOPs.
Ethiopia	Observed simulated joint team (Federal Ministry and Partners) response to the newly received information, identified key activities and assigned players	The simulated team was not able to adjust the entire outbreak response plan in favor of information given for newly identified outbreak
Kenya	Observed capacity to modify the plan with new outbreak information and response activities	The simulated outbreak plan adjusted to new outbreak information was not comprehensive enough to address all vulnerable groups. The national plan was not addressing such strategies
Malawi	Partners engaged by MOH in the outbreak including WHO, UNICEF, MCSP/ONSE to support outbreak response in the simulation exercise.	The observed existence of a limited capacity to modify the initial plan with the new outbreak information.
Namibia	Observed communication and coordination of the outbreak management team in responding to new outbreak information	The observed existence of a limited capacity of the team to modify the initial plan in responding to the new outbreak information in line with the WHO POSOPs
South Sudan	The team managed to simulate in modifying the outbreak response plan to fit the new information of the new outbreak within the country context	Observed limited inter-country key partners synchronization of efforts to support outbreak response activities simulation and delayed the decision making for human resources deployment because it was not clearly shown on the nation outbreak preparedness and response plan.
Tanzania	All subcommittees were able to address new outbreak information and prepared a response plan	The team did not link the new outbreak information to modify the previous outbreak response plan.
Uganda	The team managed to simulate a modified plan to include the new outbreak information for surveillance, communication, and vaccination responses.	The plan missed a vaccine withdrawal plan and mechanism for funds mobilization.

**Table 7:** the overall ability of the country to respond to an outbreak

Country	Observed strengths	Observed weaknesses
Eritrea	The observed lead role of the national task force in responding to an outbreak	Observed limited technical capacity to respond to polio event or outbreak and the National Outbreak response plan needs revision in line with the WHO POSOPs and IHR 2005 guideline.
Ethiopia	The technical ability to respond to any polio event and outbreak based on the participant's knowledge and experiences	Limited observed alignment of the national polio outbreak and response plan and the IHR-2005. Observed limited familiarization of national polio outbreak among participants
Kenya	The technical ability to respond to any polio event or outbreak and rich outbreak experiences was observed in the simulation exercise.	Limited linkage of technical and rick outbreak practical experiences from the participants to the National Polio Outbreak Preparedness and Response Plan, and the WHO POSOPs were observed.
Malawi	The observed lead role of the Ministry of Health for outbreak responses.	Observed inadequately prepared /limited capacity of the technical team to respond to any polio event/outbreak in line with the WHO POSOPs.
Namibia	Observed sufficient human resource capacity in responding to polio outbreak or event	Observed limited awareness of the team on the national polio outbreak preparedness and response plan. The national outbreak and response plan inadequately aligned with the WHO POSOPs.
South Sudan	The technical ability and capacity to respond to any polio event or outbreak were observed to participants and key stakeholders involved in the polio outbreak simulation exercise.	Limited reflection of the technical outbreak practical experience from the simulation exercise participants to the National Polio Outbreak Preparedness and Response plan was observed.
Tanzania	Observed availability of the technical ability to respond to any polio event and outbreak.	The national polio outbreak response plan was not updated in line with the new version of the WHO POSOPs.
Uganda	Observed technical ability and capacity to respond to any polio outbreak or event among participants and key stakeholders participated in the simulation exercise.	The national Polio Response Plan is in line with the WHO POSOPs with gaps in the details of sub-committee's membership, task, and roles.

## Discussion

We observed that the polio outbreak response simulation exercises conducted in the 8 countries had an orientation session to participants before the real exercise. The orientation session capacitated participants with basic outbreak response knowledge and the ability to simulate the outbreak response and to contribute to the further improvement of their national polio outbreak preparedness and response plans. The review realized that the conducted simulation exercises were a mix of tabletop and drill exercises. Studies have shown that these two types of simulation exercises can increase operational resilience, improve effectiveness and efficiency, and protects the organizational values in dealing with outbreaks [23,34,35]. In this regard, simulation should be conducted to countries at least after every 3 to 5 years for the improved preparedness and response to not only polio outbreaks but all VPVs outbreaks. In most of the countries that conducted simulation exercises, the outbreak communication and coordination mechanisms were not in line with IHR 2005. The awareness that polio outbreak and or polio event are among diseases of public health emergencies of international concern was limited. The conducted polio outbreak simulation exercises were used as platforms for dissemination of the IHR 2005 which supported countries in dealing with all diseases of public health emergencies. Additionally, even though less evidenced, the conducted polio outbreak simulation exercises contributed to the overall improved communication and coordination for outbreaks in these countries.

We realized the national polio preparedness and response plans were familiar to some key EPI officers while the Ministries of Health Managements teams were less informed even though endorsed the document. Surprisingly we realized in making decisions or communicating on the outbreaks, the Ministries of Health used their senior officers and not the key EPI Officers. In this regard, the simulation exercise eye opened the key stakeholders and the Ministries of Health Management of their roles and responsibilities in line with the WHO polio outbreak standard operating procedures and IHR 2005 guideline when an outbreak is being detected in the country. The initial outbreak response capacities reviewed were found to be limited in most countries except in South Sudan and Uganda where the team managed to prepare the initial outbreak response plan within the allocated simulation exercise time. However, all plans reviewed were not comprehensive to cover all at-risk groups in the country and were inadequately aligned with the standard WHO standard templates. Most of the prepared plans had no details of key activities, activities timelines, responsible persons, and were not aligned with the WHO POSOPs and IHR 2005 guideline.

It was observed in the simulation exercises; most participants were using their individual outbreak experiences rather than their national plans and guidelines. This resulted from the limited information found on the countries polio outbreak preparedness and response plan in dealing with the outbreak. Nevertheless, this was an opportunity for the teams to revise their national plans and aligned to the WHO POSOPs and IHR 2005. Interestingly, the simulated countries tried to prepare the mandatory outbreak situational report (SITREP) using the standard WHO template in the allocated time. In the outbreak, situation countries are expected to have flexible plans and especially expect either an increased number of cases of the outbreak or emerging of a new similar outbreak in different settings. We revealed that countries presented parallel plans of responding to the outbreaks in the simulation exercises. In turn, this was noted to be an expensive strategy and it was emphasized on developing flexible outbreak response plans to integrate emerged new outbreaks. We found out most countries had overall technical capacities and expertise to deal with outbreaks to a certain extent. Nevertheless, we noted that the national polio outbreak preparedness and response plans were not comprehensive enough to provide proper guidance. The guidelines were inadequately aligned with the WHO POSOPs and IHR 2005. Additionally, most participants in almost all countries were less familiar with their national polio outbreak preparedness and response plans, in particular, the management teams at the Ministries of Health and the heads of key immunization stakeholders.

## Conclusion

We still believe that the risk of a polio outbreak and other diseases to occur in East and Southern Africa countries is still high due to existing population immunity and surveillance gaps and movement of people to and from countries with polio outbreaks. We also realized that, in all countries where the polio simulation exercise conducted there was an opportunity of improving their national polio outbreak preparedness and response plan in line with WHO polio outbreak standard operating procedures and IHR 2005. The knowledge gained while conducting polio outbreak simulation exercise can also be used in responding to other vaccine's preventable diseases. We strongly believed the polio outbreak simulation exercises improved the national outbreak preparedness and response plans and we recommend the activities should be done at least every 3-5 years due to the rapid turnover of staff and to accommodate more practices following revisions of guidelines.

### What is known about this topic

- Polio outbreak simulation exercise is emphasized in the polio endgame Strategy 2019-2023, in the poliovirus containment section;
- The East and Southern Africa sub-region WHO office conducted a polio outbreak simulation exercise in some countries to increase their level of preparedness to the polio outbreaks;
- The simulation exercises are being conducted regularly to improve the capacities in the preparedness and response to various emergencies such as fire drills.

### What this study adds

- The study identifies weaknesses and recommends ways for improving future polio outbreak simulation exercises to improve national polio preparedness and response plans;
- The study provides valuable information on the conducted polio outbreak simulation exercise in the ESA countries which can be used by the Global Polio Eradication towards polio eradication and beyond polio eradication;
- The study indicates the need to inform senior management officers of key immunization stakeholders on their roles and responsibilities in the polio outbreak situations to ESA countries.
